# Short-term Intermittent Fasting for Weight Loss: A Case Report

**DOI:** 10.7759/cureus.4482

**Published:** 2019-04-16

**Authors:** Soo Liang Ooi, Sok Cheon Pak

**Affiliations:** 1 Integrative/complementary Medicine, Charles Sturt University, Bathurst, AUS

**Keywords:** weight loss, 5:2, body fat ratio, cholesterol, uric acid, single-case design, fasting

## Abstract

Intermittent fasting, in which individuals fast periodically, is an increasingly popular weight loss regimen. To understand the short-term effects of such a regimen, we present a case of intermittent fasting with data collection that mimics the single-case design.

A healthy but slightly overweight adult male underwent complete fast for two full days and resumed with normal eating for five days, and repeated the cycle three times. Data were collected from three periods: baseline (one week); fasting (three weeks); post-fasting (one week). Measurements taken daily include weight, body fat ratio, temperature, blood pressure, blood glucose, as well as waist and hip circumferences. Blood tests were conducted weekly for safety screening and to obtain observations on lipid profile, high-sensitive C-reactive protein (hsCRP), hemoglobin A1c (HbA1c), and uric acid.

The participant lost 1.3 kilograms (kg) in body weight (W̅b = 65.9kg vs W̅p = 64.6kg). Body fat ratio did not differ much (F̅Rb = 19.1% vs F̅Rp = 18.8%). Fasting caused an acute drop in the blood glucose level, which was restored upon resuming normal eating. Total cholesterol dropped drastically immediately after the first fasting cycle but rebounded 15% higher than baseline before dropping down. Fasting also temporarily raised uric acid levels, blood pressure, and body temperature. HbA1c and waist and hip circumferences were not affected by fasting. Improvement in inflammatory marker (hsCRP) was observed (2.0 to 0.3 milligrams per liter, mg/L).

This case demonstrates that intermittent fasting can induce short-term weight loss and reduce acute inflammatory marker in a healthy adult, but not body fat ratio and lipid profile. Similar single-case study design can be applied across a practice-based network for inter-case replication.

## Introduction

Intermittent fasting is an encompassing term covering any eating plan that alternates between fasting and non-fasting periods. The different types of intermittent fasting include complete alternate-day fasting, modified fasting regimens, and time-restricted feeding [1]. Intermittent fasting has become an extremely popular dietary plan and a lifestyle approach for weight loss. A search with the term “Intermittent fasting” on the Google Books online (books.google.com) returned almost 100,000 titles on this topic as of January 2019. Not surprisingly, intermittent fasting has been widely promoted on the conventional, digital, and social media with health claims such as facilitating weight and body fat loss, lowering the risks of type II diabetes and cardiovascular diseases, promoting cellular regeneration, reducing inﬂammation and oxidative stress, as well as slowing the aging process.

These claims are indeed backed up by a large body of research on animals and several human intervention studies with promising ﬁndings. A recent review of the metabolic effects of intermittent fasting found evidence that supports the practice of intermittent fasting to induce sustained improvements in human health [1]. A systematic review published in 2018 found intermittent fasting to be effective in reducing weight irrespective of body mass index. Furthermore, signiﬁcant decreases in fat mass, low-density lipoproteins, and triglycerides were consistently reported in all included studies. However, this systematic review included only four high-quality clinical trials [2]. A 2018 meta-analysis of six clinical trials also found intermittent fasting to be more effective than no treatment for weight loss but not superior to continuous energy restriction [3].

While evidence is emerging to support the beneﬁcial effects of intermittent fasting as a nonpharmacological approach to improve health, there is still much unknown with regard to the eﬃcacy of various forms of intermittent fasting, the replicability of the metabolic effects seen in animal studies to humans, and the long-term safety of energy restriction [4]. Furthermore, there is still the challenge of translating research evidence into practice without any established clinical guidelines in place.

With the increasing popularity of intermittent fasting, clinicians, especially those in primary care, will inevitably face with patients who are fascinated by the overzealous claims on the media and wish to try out intermittent fasting. Instead of dismissing it as a fad and risk having the patients undergo intermittent fasting unsupervised, a clinician can discuss with the patient on the available evidence and any potential health implication. Given that supervised fasting is safe and with only mild to moderate and known reactions, should the patient persist, the clinician can consider working with the patient to study the time course, variability, and effect of the intervention in clinical practice, if time and cost permit [5]. In this way, clinicians can gain further insights to personalize weight management strategies for the patient and to mitigate any potential health risk.

We present the results of such a case for reference, illustration, and discussion. This is a clinical case report. The individual provided his written informed consent in sharing the case history and the data collected for publication.

## Case presentation

Case history

A healthy but marginally overweight Asian male (age 48 years) was interested to explore the use of intermittent fasting as a lifestyle approach for weight loss. He was 83 kilograms (kg) previously and had successfully reduced his weight to 61 kg by adopting a strict plant-based diet and exercise over a period of three years. He was moderately active and walked for at least an hour daily with his pedometer count averaging 12,500 steps over the past two years. He was not diagnosed with any chronic conditions. He did not have any history of gastrointestinal issues other than an isolated incident of bleeding duodenal ulcer when he was a teenager, which had since been resolved after treatment with ranitidine.

Despite continuing to maintain a plant-based diet and a moderate exercise, he was experiencing gradual weight gain to 66 kg over the last two years. With a height of 1.64 meters (m), his body mass index was 24.53 kg/m^2^, putting him back to the overweight category under the World Health Organization’s obesity guidelines for Asia-Pacific populations. His waist circumference was 88.5 cm, only slightly below the abdominal obesity cut-off point for Asians (90 cm in men) [6]. With a family history of stroke, he was keen to reduce his weight back to the normal range.

Interventions and data collections

The case individual chose to pursue a variant of the popular five-two (5:2) intermittent fasting regimen (a modified fasting regimen with severe energy restriction for two days per week and ad libitum eating for the other five days). Instead of consuming 20% to 25% of energy needs on two non-consecutive fasting days per week as per the standard 5:2 protocol, the individual opted to observe complete fasting with no energy-containing foods or beverages consumed for two consecutive days per week, making the total hours of fasting being 48 hours or more but not over 60 hours. The individual had been through 18 hours of fasting as part of his religious practice before which gave him confidence that he could go on for two consecutive days without food. He also intended to maintain his normal physical activity level throughout the fasting periods as much as he could.

To assess the effectiveness of this weight loss plan and its metabolic effect, the individual consented to take daily measurements on weight, body fat mass and ratio, temperature, blood pressure, fasting blood glucose, as well as waist and hip circumferences. All measurements were taken using household health monitoring equipment (Table 1) upon waking before consuming any food or drinks. Daily physical activity level (measured in the total number of steps) was also monitored using a pedometer worn during waking hours. The individual also agreed to undergo blood tests weekly at a commercial medical laboratory for safety screening and to obtain observations on lipid profile, high-sensitive C-reactive protein (hsCRP), hemoglobin A1c (HbA1c), and uric acid. During the fasting period, blood tests were taken in the morning after two consecutive days of fasting before any meal.

**Table 1:** Equipment used for daily measurement kg, kilograms; mmHg, millimeters of mercury; mmol/L, millimoles per liter; ^o^C, degrees Celsius

Equipment	Brand & Model	Specification
Body composition monitor	Tanita Inner Scan BC-541	Max: 150kg, Increment: 0.1kg; Fat % Increment: 0.1%.
Automatic blood pressure monitor	Omron HEM-7200-C1	Pressure: 0 mmHg to 299 mmHg. Accuracy: ±3 mmHg.
Blood glucose meter	i-sens CareSens N GM505PAB	Range: 1.1-33.3 mmol/L. Standard Deviation: 0.1 mmol/L. Coefficient of Variation: 3.6%.
Infrared forehead/ear thermometer	Guardian FET1C	Range: 32.0 to 42.9. ±0.2 ^o^C
Waist and hip measuring tape	N/A	Max 60 inches or 1.5 meters (has both inch and metric graduations on both sides).
Pedometer	Actxa Swift Activity Tracker	Steps tracking with 3-axis accelerometer and vibration motor

For safety, the individual was encouraged to drink enough water to avoid dehydration. He was to observe symptoms including irregular heart rhythm, sweating, shakiness, anxiety, excess hunger, and nausea. To prevent hypoglycemia, he was told to drink one to two tablespoons of honey in warm water every 15 minutes until the symptoms subsided. Additional blood glucose test should be taken to ensure the blood glucose level did not drop below 3.0 mmol/L. He was told to stop fasting and seek medical help if the symptoms kept returning and blood glucose maintained at sub-3.0 mmol/L level. He could also choose to abandon fasting at any time.

Data were collected over three periods of five weeks (35 days): baseline (one week); fasting (three weeks); post-fasting (one week). The duration was determined by the case individual as he intended to use the fasting regimen as a short-term weight maintenance approach. Comparison of the seven-day averages of the post-fasting daily measurements of weight and body fat to the baseline averages was used to determine the effectiveness of the diet plan. The individual was also advised to keep a journal on his subjective experience of fasting. We used R Studio Version 1.1.453 running on R version 3.5.1 for visual data analysis.

Results

Body Weight

**Figure 1:**
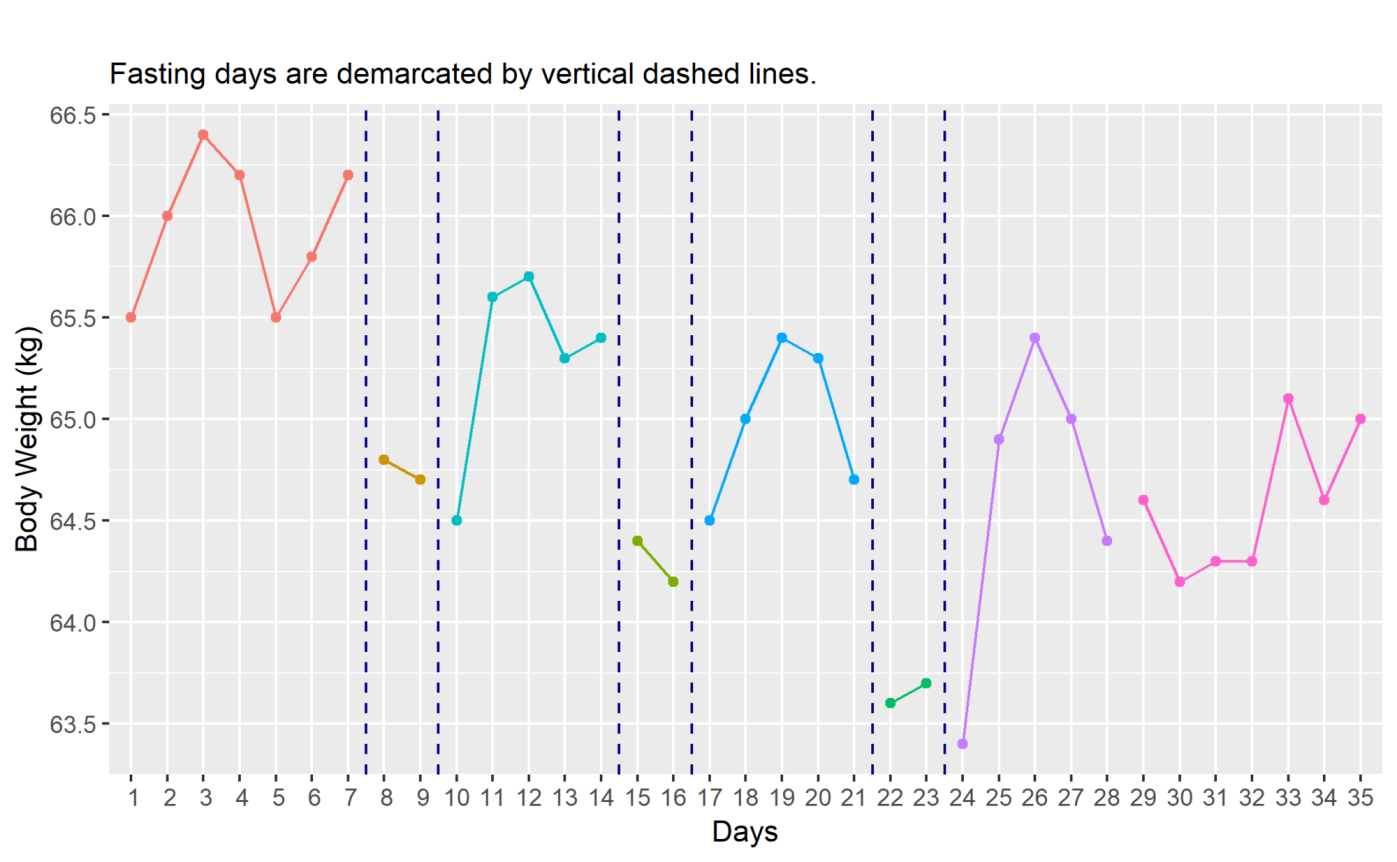
Time course of changes in body weight (daily) kg, kilograms

The time course of the body weight changes is shown in Figure 1. At baseline, the daily weight measurements range between 65.5 kg to 66.4 kg with a mean of 65.9 kg (W̅b). An immediate drop in weight is noticeable on two consecutive fasting days and the drop continues one more day with resumption of eating before a rebound on the subsequent days. After three cycles of intermittent fasting, weight measurements on the post-fasting week settle between a lower range of 64.2 kg to 65.1 kg with a mean of 64.6 kg (W̅p). A reduction of 1.3 kg (W̅b - W̅p) is observed, representing a 2% loss of initial body weight. 

Body Fat Ratio and Mass

**Figure 2:**
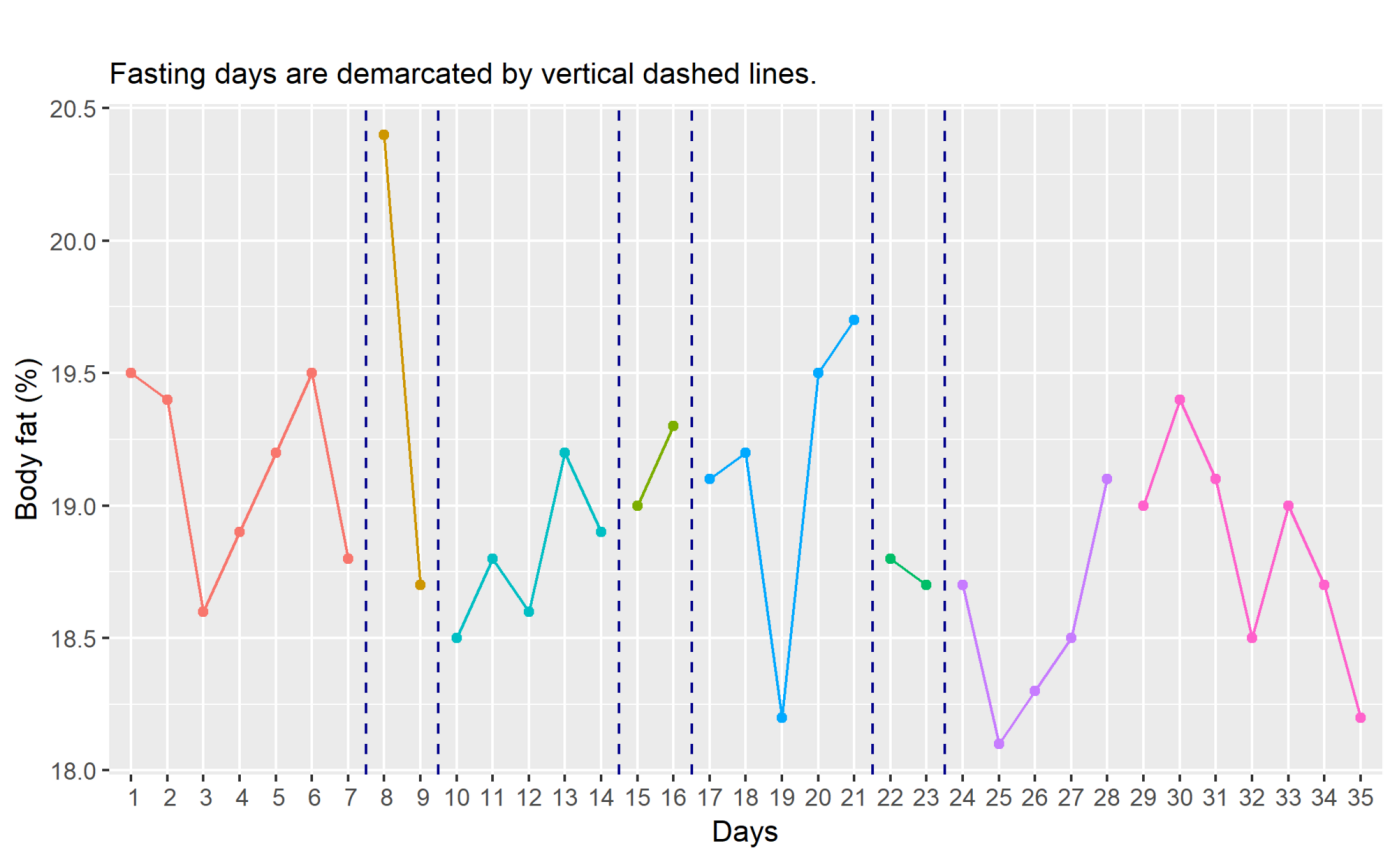
Time course of changes in body fat ratio (daily)

Unlike changes in the body weight over time, no clear pattern emerges from the visual analysis of body fat ratio measurements, as shown in Figure 2. The baseline average of body fat ratio (F̅Rb) is 19.1% (Range: 18.6% to 19.5%). In comparison, the post-fasting average body fat ratio (F̅Rp) is 18.8% (Range: 18.2% to 19.4%). This change in body fat ratio (F̅Rb - F̅Rp = 0.3%) is considered too small to be of any clinical significance.

**Figure 3:**
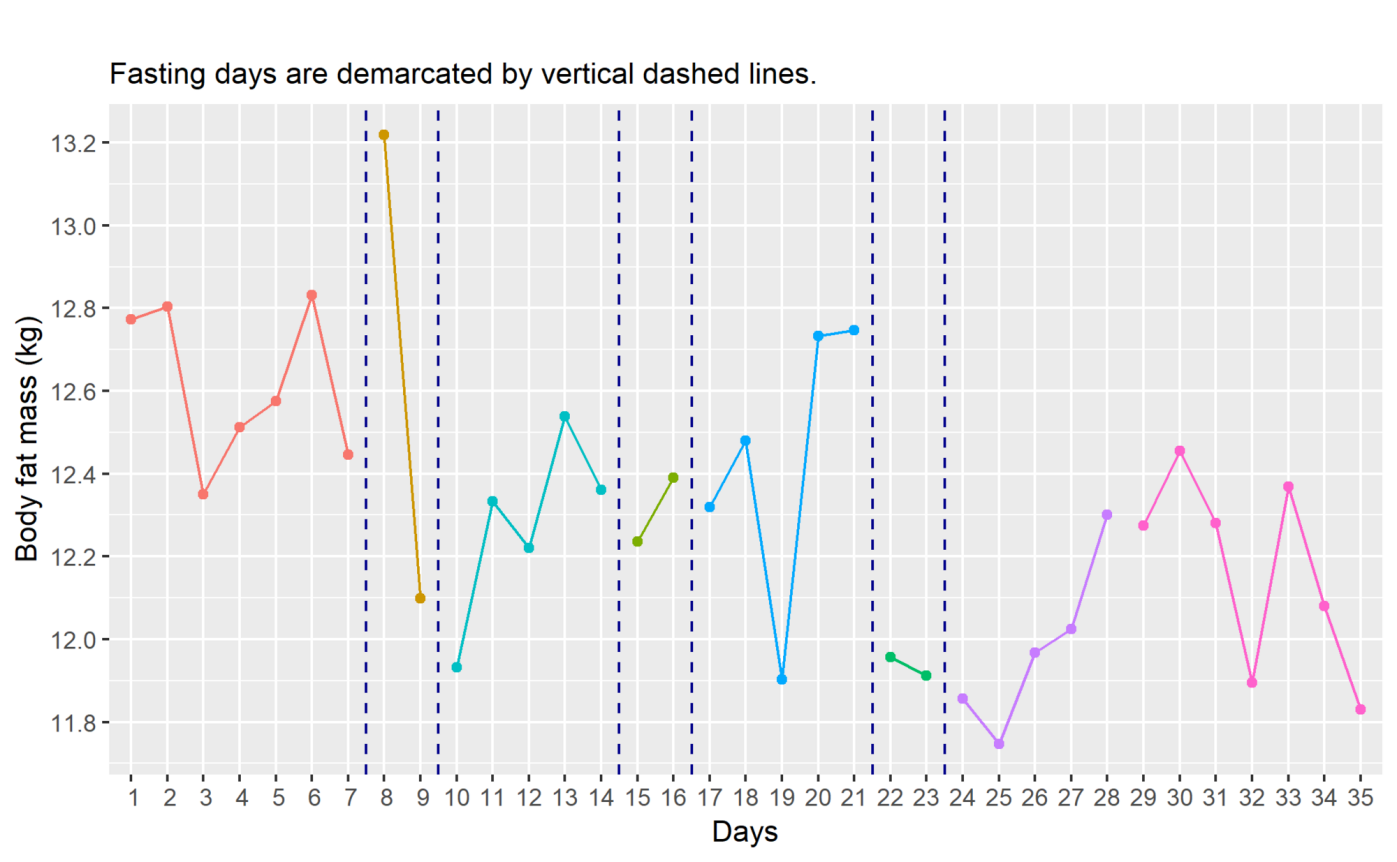
Time course of changes in body fat mass (daily) kg, kilograms

The time course of the changes in body fat mass which closely follows the changes in body fat ratio is shown in Figure 3. The initial average body fat mass (F̅Mb) is 12.61 kg (range: 12.35 kg to 12.83 kg), and the post-fasting average of body fat mass (F̅Mp) is 12.17 kg (range: 11.83 kg to 12.45 kg). A drop of 0.44kg (F̅Mb - F̅Mp), which is about 3.6% of initial body fat mass, is detected. 

Physical Activity

**Figure 4:**
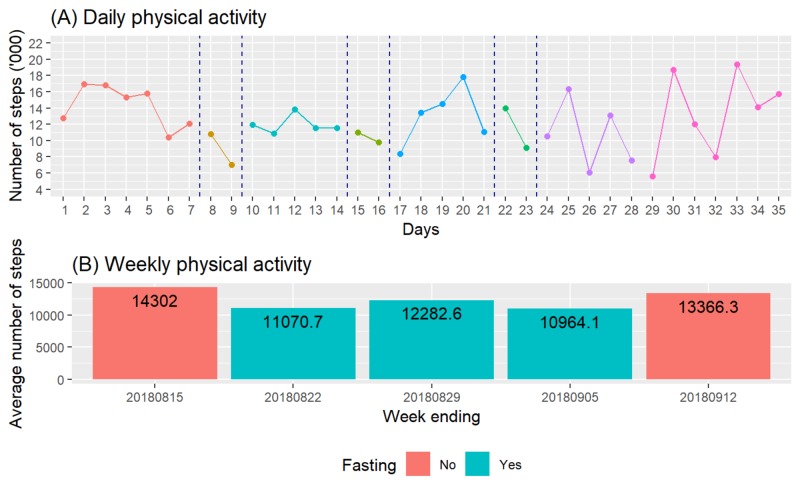
Time course of changes in physical activity

Figure 4A shows the daily physical activity measured in the number of steps recorded. Although the individual intended to maintain his normal physical activity level throughout the fasting period, there appeared to be a drop in physical activity levels during fasting days. However, we cannot derive any clear pattern since there he also recorded lower physical activity levels during several non-fasting days. Figure 4B compares the average numbers of steps per week over five periods. It appears that the individual naturally reduced his physical activity levels during fasting periods. Hence, any changes in body weight and fat cannot be a result of any increased physical activity level.

Blood Glucose

**Figure 5:**
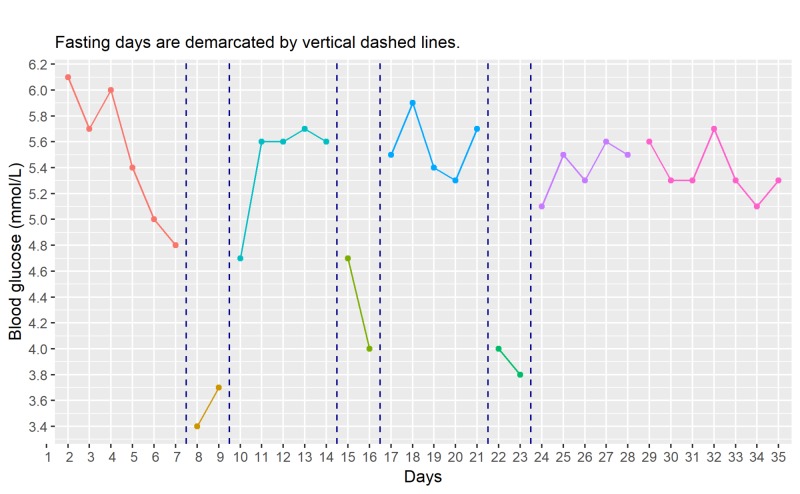
Time course of changes in fasting blood glucose (daily) mmol/L, millimoles per liter

The daily fluctuations in fasting blood glucose level are shown in Figure 5. Barring for a seemingly downward trend during the baseline period before the first fasting week, fasting blood glucose level appears to flicker around 5.0 to 5.8 millimoles per liter (mmol/L). The initial downward trend during the baseline, while intriguing, is not of any clinical significance since such fluctuation is well within the normal range.

A drastic drop in fasting blood glucose levels is observed at the start of each fasting period, especially during the first fasting period which measures at 3.4 mmol/L. With the resumption of eating, the fasting blood glucose level quickly restored to the previous non-fasting level. This observation is consistent for a normal non-diabetic individual.

Other Daily Measurements

Measurements of body temperature with mean of 35.78 degrees Celsius (°C) and range of 35.3 to 36.4 °C, systolic blood pressure with mean of 101.24 millimeters of mercury (mmHg) and range of 89 to 111 mmHg, as well as diastolic blood pressure with mean of 60.24 mmHg and range of 52 to 72 mmHg, taken throughout the observation period are well within the normal ranges. Visual analysis of the data pattern over time does not reveal any effect of fasting on these parameters. Measurements of the waist and hip circumferences also show no detectable change. 

Lipid Profile

**Figure 6:**
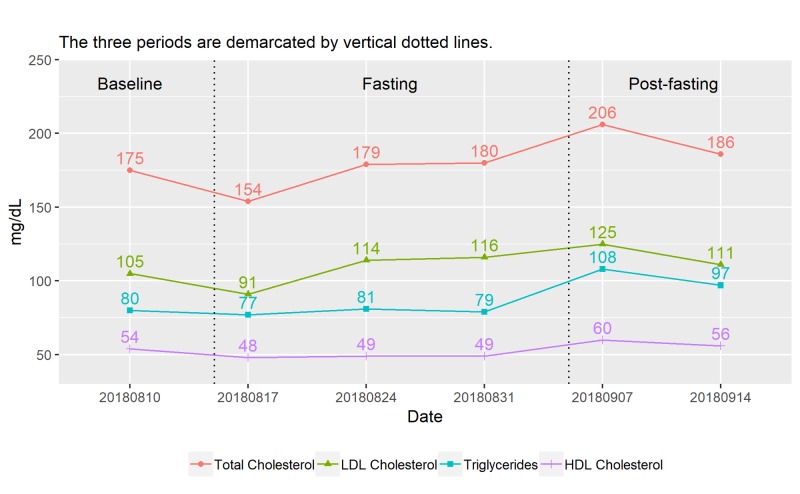
Time course of changes in lipid profile (weekly) HDL, high-density lipoprotein; LDL, low-density lipoprotein; mg/dL, milligrams per deciliter

Figure 6 shows the changes in lipid profile over time. A drop in all lipid profile parameters is observed after the first two consecutive days of fasting. However, total cholesterol level appears to rebound and rise after subsequent cycles of fasting and reach its peak at 206 milligrams per deciliter (mg/dL) one week after the last two consecutive fasting days before dropping back to 186 mg/dL, a level higher than 175 mg/dL at baseline. Hence, multiple cycles of complete 5:2 fasting appear to raise the total cholesterol level from the ideal range to the range of borderline high (200 and 239 mg/dL).

**Figure 7:**
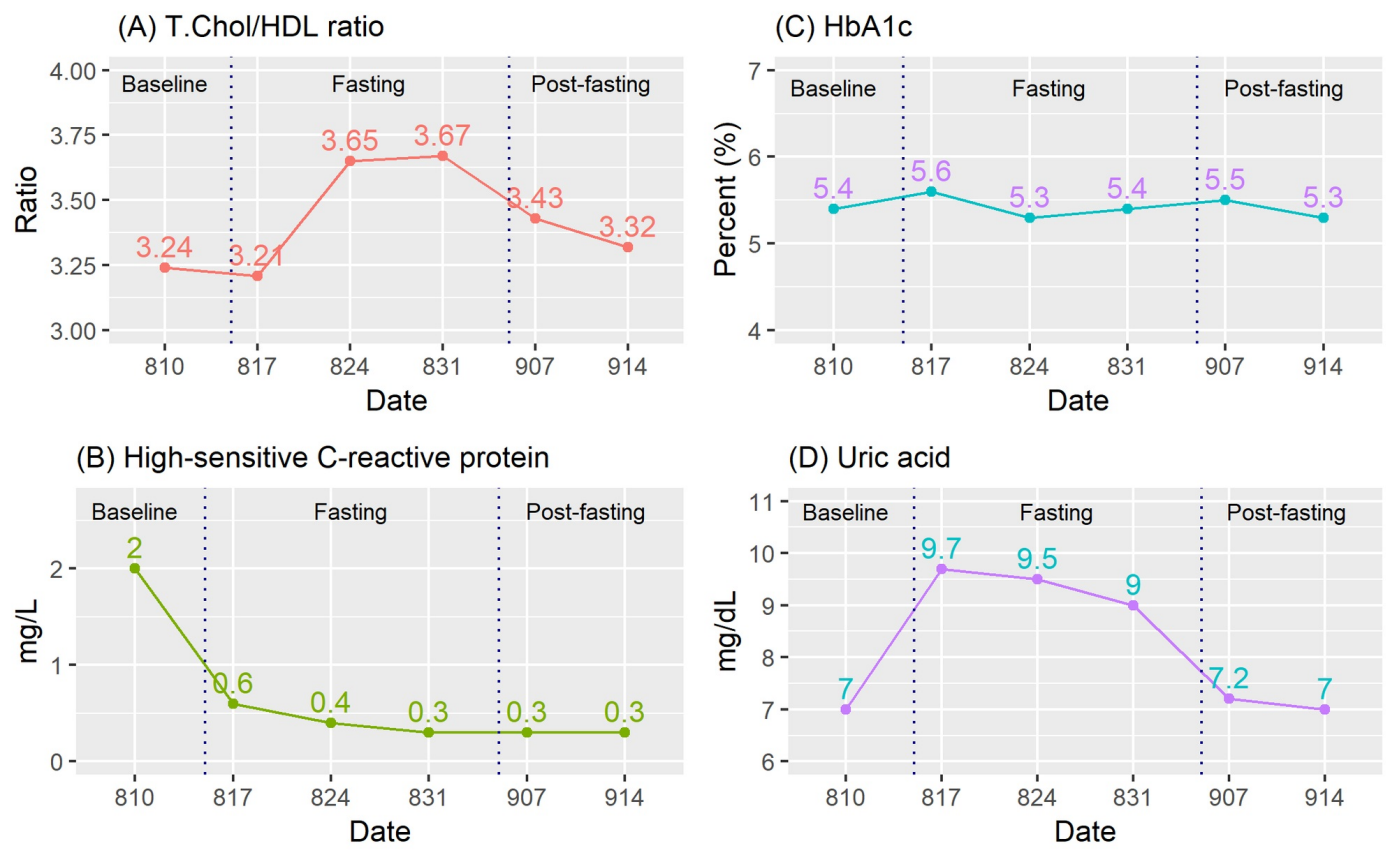
Time course of changes in other test results (weekly) HbA1c, hemoglobin A1c; HDL, high-density lipoprotein; mg/dL, milligrams per deciliter; mg/L, milligrams per liter; T.Chol, total cholesterol

The fluctuations in total cholesterol level are due mainly to the changes in the low-density lipoprotein (LDL) cholesterol and triglycerides as depicted in Figure 6. The high-density lipoprotein (HDL) cholesterol appears to be depressed by fasting, as a result, the total cholesterol to HDL ratio rises after the last two fasting cycles (Figure 7A). 

Despite the fluctuations in parameters, the subject’s lipid profile remains at the healthy level at post-fasting. 

High-sensitive C-reactive Protein, Hemoglobin A1c, and Uric acid

The inflammatory marker hsCRP is reduced drastically from 2.0 milligrams per liter (mg/L) at baseline to 0.6 mg/L after the first two consecutive days of fasting, as shown in Figure 7B. It drops further in the next two cycles and consistently stays at 0.3 mg/L at post-fasting.

Contrary to hsCRP, HbA1c is not affected by intermittent fasting as it stays consistently within the range of 5.3% to 5.6% throughout (Figure 7C). This longer-term glycemic control measure remains unchanged by short-term intermittent fasting.

The level of uric acid is another biomarker that is clearly affected by the practice of intermittent fasting. As shown in Figure 7D, the uric acid level rises from 7.0 mg/dL to over 9.0 mg/dL during the fasting periods and drops back to 7.0 mg/dL post-fasting. Fasting pushes the serum uric acid level beyond the normal range.

Adverse Effect and Subjective Experiences

All blood tests show no abnormality or drastic change in liver profile and complete blood count throughout three periods.

The case individual reported no serious adverse event and no gastrointestinal complaint. His fasting journal contained entries of “harder to get into sleep than normal” and “interrupted sleep” on the second day of fasting and feeling “weak and dizzy when first stood up upon waking” during the first fasting cycle. He also described the feeling of “mild tightness at the head akin to the experience at high altitude”. These were symptoms of mild hypoglycemia as his blood glucose level was reaching a low of 3.4 mmol/L (Figure 4). As advised, the individual drank one glass of honey water as a precautionary measure and the symptoms dissipated. He did not find it necessary to monitor his blood glucose level again. During the next two cycles, these experiences subsided as the body “must have gotten used to it”. The experience of “harder to get into sleep than normal” remained through the three cycles though.

Overall, the subject was satisfied with the results of intermittent fasting and would consider doing it again.

## Discussion

We know very little about the short-term effect of intermittent fasting as a weight maintenance approach on healthy nonobese subjects in the literature. Most intermittent fasting studies have study durations of 3 months or more to study chronicity. Heilbronn et al. reported a significant mean loss of 2.5 ± 0.5% of initial body weight and a significant reduction of the initial fat mass with a mean of 4.0 ± 1% among 16 nonobese participants (50% male) after 21 days of alternate day fasting with no caloric intake every other day [7]. Such findings are consistent with our observations in this case with the loss of approximately 2% of initial body weight and 3.6% of initial fat mass after three weeks of intermittent fasting with no caloric intake on two consecutive days per week. The weight loss is small as the individual has already lost significant weight in the past. His metabolic rate has adjusted to his current weight, making it hard to achieve drastic weight loss in just 35 days.

However, in another observation study, Halberg et al. reported no significant change in mean weight of eight healthy males was found after undergoing intermittent fasting every second day for 20 hours over a duration of 15 days [8]. Similarly, Soeters et al. found no differences in body weight of eight healthy subjects in a cross-over trial comparing alternate day fasting to the standard diet over two weeks [9]. Hence, we postulate that for intermittent fasting to be effective in achieving about 2% of weight loss in a nonobese subject, a minimum duration of three weeks is needed. While a 5% reduction in weight is commonly considered as clinically significant for the prevention and treatment of obesity, significant health benefits are achieved in association with very modest (less than 3%) weight loss in adults who adopt and sustain physical activity combined with a healthful diet [10]. Hence, three weeks of intermittent fasting can be another option for short-term weight maintenance intervention for nonobese individuals in addition to physical exercise and proper diet.

Fasting is known to induce acute changes in blood glucose level. Horne et al. [11] observed a significant mean reduction of 0.444 ± 0.854 mmol/L in 30 participants (66.7% female) after one day of water-only fast and a rebound of 0.327 ± 0.921 mmol/L after eating was resumed. In our case, the pattern of acute drop and rebound is similarly observed and the blood glucose level maintains at the lower level on the second day of fasting. It also appears that the reduction in blood glucose level is not as drastic in the second and third cycles of fasting, signifying the ability of the body to adapt to the fasting condition. Further studies with more subjects are needed to confirm this glucoregulatory observation. When comparing mean difference in post-fasting blood glucose level to that of the baseline after three weeks of intermittent fasting, we do not find any difference in this case which is consistent with other intermittent fasting studies of similar duration [7-9]. 

A review by Santos and Macedo found most intermittent fasting studies reported improvement in lipid profile including reducing total cholesterol, LDL, triglycerides and increasing HDL levels [12]. In contrast, in our case, reduction in all lipid profile parameters appears to be merely an acute reaction of the body to the impact of the first two consecutive days of fasting. Elevated levels of total cholesterol, LDL, and triglycerides during the latter part of the fasting period is detected. This can be due to individual variability or the effect of complete 5:2 fasting. Since current evidence is based mostly on the alternate day fasting or time-restricted feeding (including Ramadan fast), there is insufficient understanding on the effect of intermittent fasting on lipid profile with more than 24 hours of complete energy restriction. This can be an area of further research.

Weight loss is known to associate with a decline in the level of C-reactive protein (CRP), an inflammatory marker implicated in the risk of developing chronic diseases including cardiovascular disease, diabetes, and cancer [13]. Surprisingly, the findings from intermittent fasting studies were mixed with some studies found significant improvements in inflammatory markers including CRP while others did not [1]. In the current case, the changes in the hsCRP as an effect of intermittent fasting is remarkable (from 2.0% to 0.3%). However, we should interpret this observation with caution and not generalize. hsCRP is an acute phase reactant of the inflammatory response which is affected by many conditions. It is not a measure for the risk of inflammation. Furthermore, the body may synthesize less CRP in fasting due to the lack of protein sources. Hence, any change in CRP does not necessarily represent a change in initial clinical state as other inflammatory processes may affect the CRP values. More research on the effect of intermittent fasting on hsCRP is required.

In overweight and obese subjects as well as patients with type II diabetes, intermittent fasting was equally effective in improving longer-term glycemic control indicator measured by the HbA1c within a 12-month period, compared to continuous energy restriction [14-15]. However, in studies of shorter duration and in non-diabetic population, reduced HbA1c has not been consistently demonstrated [1,16]. As HbA1c is reflecting the cumulative glycemic history of the preceding two to three months, the fluctuation in blood glucose level caused by short-term intermittent fasting has no effect on this marker as shown in the present case. Hence, HbA1c is not an appropriate measure here. The 1,5-anhydroglucitol test can be a better marker for short-term glycemic control to study the effects [17].

We notice the rise of serum uric acid during fasting. This is to be expected. During fasting, the body uses other stores for energy which include the breakdown of stored proteins/amino acids and fats. Uric acid is a waste product of this catabolic process. Fasting has been reported to increase uric acid in the literature. Gumaa et al. reported the linear increase of serum uric acid level with the duration of Ramadan fast among 16 volunteers [18]. Runcie and Thomson also found the occurrence of hyperuricemia in 42 obese patients treated with total fasting [19]. However, the effect was apparently harmless as none of the patients develop acute gout. 

This case report mimics the single subject research design which can be a useful tool in practice-based primary care research [20]. The repetitive cycle of intermittent fasting makes it natural for observations to be made with multiple periods of baseline (i.e. non- fasting days) and multiple times for intervention (i.e. fasting days) as per the primary A-B single-subject design. While observations from a single case cannot be generalized, the same methodology can be replicated across a practice-based network for further understanding of the effects of intermittent fasting on different subjects. Collection of cases can be used for meta-analysis and inferential statistical tests, advancing the knowledge in the field. 

## Conclusions

This case demonstrates that three weeks of intermittent fasting can induce short-term weight and fat mass loss with a reduction in acute inflammatory marker in a healthy adult, but not body fat ratio and lipid profile. Complete fasting for two full days per week is well-tolerated by the individual. Although improvement in lipid profile through intermittent fasting is commonly reported in the literature, this case shows a temporary rise in lipid profile parameters during fasting even though the effect seems to be transient. Transient elevation of serum uric acid level is also observed. Similar single-subject study design can be applied across a practice-based network for inter-case comparison and analysis.
